# A surface-enhanced Raman scattering-based approach for rapid and highly sensitive quantitative analysis of 3-carboxy-4-methyl-5-propyl-2-furanpropionate and indole-3-acetic acid in saline, human serum and uremic serum of patients with chronic kidney disease

**DOI:** 10.1039/d0ra06123a

**Published:** 2020-12-08

**Authors:** Shaghayegh Saadati, Ubong Eduok, Amira Abdelrasoul, Ahmed Shoker

**Affiliations:** Department of Chemical and Biological Engineering, University of Saskatchewan 57 Campus Drive Saskatoon Saskatchewan S7N 5A9 Canada amira.abdelrasoul@usask.ca +306 966 4777 +306 966 2946; Division of Biomedical Engineering, University of Saskatchewan 57 Campus Drive Saskatoon Saskatchewan S7N 5A9 Canada; Nephrology Division, College of Medicine, University of Saskatchewan 107 Wiggins Rd Saskatoon SK S7N 5E5 Canada; Saskatchewan Transplant Programn, St. Paul's Hospital 1702 20th Street West Saskatoon Saskatchewan S7M 0Z9 Canada

## Abstract

3-Carboxy-4-methyl-5-propyl-2-furanpropionate (CMPF) and indole-3-acetic acid (IAA) are critical protein-bound uremic toxins that occur during chronic kidney disease (CKD). This study offers the first reported instance of surface-enhanced Raman scattering (SERS) coupled with an Au nanoparticle substrate for the simple quantification of CMPF and IAA in human serum samples. The detection limits of the CMPF and IAA analysis were estimated to be 0.04 nM (S/N = 3) and 0.05 μM (S/N = 3), respectively. The results demonstrate that the SERS technique is fast-acting and highly sensitive when it comes to the simultaneous and individual quantitative detection of CMPF and IAA in biological samples. We believe that this analytical tool could serve as a very useful method for practical applications during the analysis of CMPF and IAA in the serum and urine of patients at all stages of CKD and of healthy volunteers as well as in various reservoirs.

## Abstract 2

Rapid and highly sensitive quantitative analysis of 3-carboxy-4-methyl-5-propyl-2-furanpropionate and indole-3-acetic acid in uremic serum of patients with chronic kidney disease.

## Graphical abstract

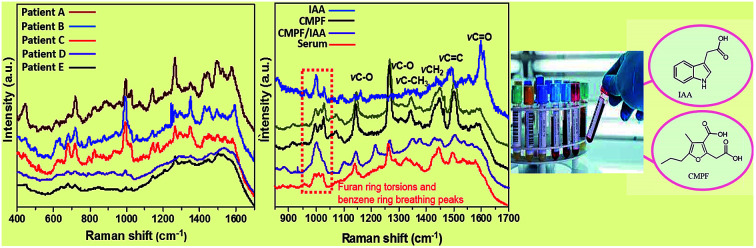

## 1. Introduction

Chronic kidney disease (CKD) is an emerging world health problem.^1^ CKD disturbs the human metabolism as a disease state called uremia.^2^ In patients with CKD, uremic toxins accumulate in the blood and their levels are a predictor of cardiovascular events and mortality.^3^ Uremic toxins are correlated with and identified as inducers of oxidative stress, inflammation, and endothelial dysfunction.^4,5^ One class of uremic toxins is protein-bound uremic toxins (PBUTs) that mainly bind with human serum albumin (HSA) and are not removed by conventional hemodialysis and is of major concern.^6,7^ 3-Carboxy-4-methyl-5-propyl-2-furanpropanoic acid (CMPF) and indole-3 acetic acid (IAA) are critical PBUTs. CMPF is a metabolite of furan fatty acid and a marker of fish oil intake and it is almost 100% bound to protein. CMPF interacts with free oxygen radicals, which can induce cell damage, and it can inhibit tubular secretion, drug metabolism in the liver; the uptake of erythromycin by inhibiting Oatp2, hepatic uptake, and/or efflux transporter. Also, CMPF leads to the inhibition of deiodination of thyroxine (T3) by inhibiting drug binding to some proteins.^8,9^ IAA is a uremic indolic toxin derived from the metabolization of dietary tryptophan by the gut microbiota. IAA increases the endothelial expression and procoagulant activity of tissue factor (TF), increases the mRNA expression of the enzyme cyclooxygenase-2 (COX-2), which is primarily responsible for the synthesis of inflammatory prostanoids,^10^ the principal initiator of blood coagulation.^11^ Moreover, IAA is involved in the progression of interstitial renal fibrosis. *In vitro*, IAA induces free radical production in tubular cells, activates NF-kappa B and PAI-1 promoter, and increases PAI-1 expression.^12^

The determination of these compounds on a repeated basis during a dialysis session would provide a greater understanding of the CKD pathology and a diagnostic strategy for predicting disease progression and complications. Also, monitoring the PBUT concentration in serum samples is important to compare the efficiency of therapeutic strategies that have been reported to decrease their plasma concentration from the bloodstream of CKD patients. Several chromatography-based analytical methods have been used for detecting PBUTs.^13–21^ Although these methods have high separation power, sensitivity and/or selectivity, the HPLC and HPLC-coupled analyses have several limitations such as requiring sophisticated analytical chromatography systems and expertise, complicated sample preparation, including grinding, extraction, elution and sometimes heat/acid precipitation, cumbersome analysis process and time-consuming (not suitable for rapid field testing at the point of sale).

Therefore, there is a demand to develop new, simple techniques that can overcome the above-mentioned disadvantages and act with high specificity, sensitivity and reproducibility for the reliable determination of PBUTs in biological samples.

Surface-enhanced Raman scattering (SERS) is a powerful technique for biochemical analyses because of its high molecular specificity (fingerprint information on biological systems), high sensitivity (even down to the single-molecule level), simplicity, biocompatibility, and multiplexing capability with single wavelength excitation.^22^ SERS combine the fingerprint information of chemical compounds on Raman spectroscopy with high sensitivity gained by signal enhancers (plasmonic substrates).^23^ Furthermore, SERS-active nanostructures can be designed and modified for different determination purposes. To the best of our knowledge, there has been no reports demonstrating the use of SERS techniques for the analysis of PBUTs in biological samples. Herein, we present for the first time the simultaneous and individual determination of CMPF and IAA in saline and human serum samples.

## 2. Results and discussions

### 2.1. Detection and quantification of CMPF and IAA in saline media

The slide format of Au nanoparticle SERS substrates was utilized in this study for the detection and quantification of CMPF and IAA in saline as a model matrix.

**Fig. 1:**
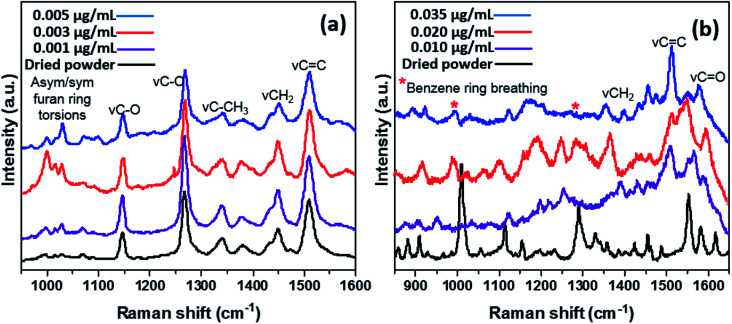
SERS spectra of different concentrations of CMPF (a, 0.001, 0.003 and 0.005 μg mL^−1^) and IAA (b, 0.010, 0.020 and 0.035 μg mL^−1^) in simulated saline media compared to their pure powdery samples.

Fig. 1a depicts the SERS spectra of CMPF molecules with four dominant peaks at 1150, 1275, 1450 and 1510 cm^−1^, corresponding to stretching vibrations of the C–O bond, CH_2_ deformations and C=C bond. There are common Raman peaks for all CMPF concentrations (0.001, 0.003 and 0.005 μg mL^−1^ CMPF) within the saline solution under the study compared to those for the dried CMPF reference powder. Apart from the prominent peaks, there are also bands attributed to asymmetric and symmetric furan aromatic ring torsions between 1000 and 1050 cm^−1^ as well as *ν*C–CH_3_ vibrations (associated with the CH_3_ umbrella mode) around 1325 and 1375 cm^−1^. The presence of AuNPs induced the observed SERS signals due to their inherent inter-particulate gaps; closer gaps generate hot spot effects.^24^ It is noteworthy to mention that the peak intensity increased as the CMPF concentration in the saline solution increased from 0.001 to 0.005 μg mL^−1^. Fig. 1b shows the Raman spectra of the IAA powder as a reference sample and its different concentrations (0.01, 0.02, and 0.035 μg mL^−1^) in saline. There are several characteristic Raman bands for IAA at 1574 cm^−1^ representing the *ν*C=C vibrations at 1554 cm^−1^ attributed to the pyrrole's C–C stretching vibrations and at 1454 cm^−1^ due to *γ*C=C (in-plane) vibrations. The peak at 1431 cm^−1^ arises from the NCC stretching for the NH bond. The peak at 1360 cm^−1^ is attributed to the Fermi doublet bond. The 1010 cm^−1^ band is due to a benzene ring breathing mode. The doublet at 1220 cm^−1^ and 1240 cm^−1^ can be attributed to CH and NH bend modes. The NH bend band also occurs at 881 cm^−1^. The singlet band at 921 cm^−1^ belongs to the *γ*OH vibration.

### 2.2. Detection of CMPF and IAA in real serum samples of healthy subjects

**Fig. 2:**
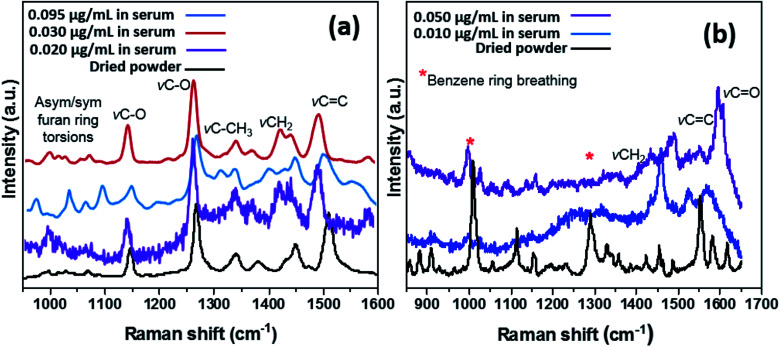
SERS spectra of different concentrations of CMPF (a, 0.020, 0.030 and 0.095 μg mL^−1^) and IAA (b, 0.010 and 0.050 μg mL^−1^) in real serum samples compared to their pure powdery samples.

**Fig. 3:**
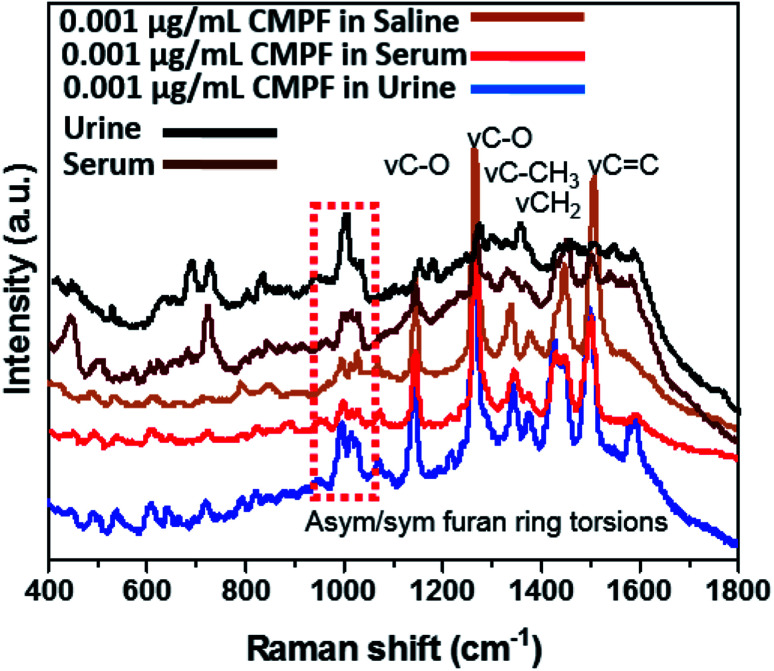
SERS spectra of 0.001 μg mL^−1^ of CMPF in the urine sample and 0.001 μg mL^−1^ of CMPF in the serum sample in compared with 0.001 μg mL^−1^ CMPF in saline solution in the range of 400–1800 cm^−1^.

In order to determine the capacity of the SERS substrate to detect these two analytes within CMPF and IAA in a more complex mixture, Raman measurements were conducted in serum after spiking the samples with aliquots of the analyte molecules. Measurements with each sample were performed with the laser power of approximately 3.0 mW and an excitation wavelength of 514.5 nm radiation from an argon-ion laser similar to the last experimental run. As expected, Fig. 2a shows the four predominant SERS peaks consistent with the CMPF molecule at 1150 and 1275 cm^−1^ (C–O bond stretching vibrations), 1450 cm^−1^ (CH_2_ deformations) and 1510 cm^−1^ (C=C bond). Relative to the SERS spectrum of the pure powder, the spectra for all CMPF concentrations (0.020, 0.030 and 0.095 μg mL^−1^ CMPF) within the serum samples showed common bands. There are still peaks consistent with the furan ring torsions and *ν*C–CH_3_ vibrations at around 1000–1050 cm^−1^ and 1325–1375 cm^−1^, respectively. There is a small shift of 5 cm^−1^ observed in the CH_3_ and CH_2_ deformation of the CMPF in serum samples when compared to the saline solution. The observed spectra were collected from healthy serum samples spiked with CMPF. Unlike CMPF, there are some observed matrix-based interferences influencing the IAA SERS signals due to the complex serum solution (see Fig. 2b). This anomaly could have been primarily due to preferential surface crowding by other molecules within the complex serum mixture.^25^ However, there are still peaks consistent with *ν*CH_2_, *ν*C=C and *ν*C=O vibrations at 1450, 1510 and 1620 cm^−1^, respectively. There are also peaks related to the ring-breathing aromatic benzene mode for the IAA moiety at 992 and 1299 cm^−1^. It is worthy to mention that the intensities of prominent peaks increased with IAA concentrations. A summary of vibrational normal modes and their correlation to the structure in the Raman spectra of CMPF and IAA is presented in Table 1 and 2. Due to the satisfactory performance of CMPF and IAA in the saline solution, we continued to evaluate the feasibility of this method for CMPF and IAA detection in healthy urine and serum samples. Fig. 3 shows the Raman spectrum of CMPF (0.001 μg mL^−1^) in saline, serum and urine samples in the Raman shift range of 400–1800 cm^−1^. The protein interfaces between 500 and 1000 cm^−1^. As presented, in Table 1 and 2, the SERS approach was less susceptible to interference from the serum and urine matrix.

**Table 1:** Raman bands of the CMPF in saline and serum samples from healthy subjects

Description of CMPF peaks	Raman shift (cm^−1^)			
	Powder sample	Saline	Serum	Urine
CH_3_ and CH_2_ deformation	1455	1455	1440	1458
CH_3_ and CH_2_ deformation	1465	—	—	—
CH_3_ umbrella mode	1380	1380	1380	1380
Stretching vibrations of the C–O bond	1260	1260	1260	1260
C–O	1170	—	—	—
C–C	1020	1020	1020	—
Symmetric furan ring torsion	1000	1000	1000	1000–1050
C=C in the furan ring	—	—	—	1600

**Table 2:** Raman bands of the IAA in saline and serum samples from healthy subjects

Description of IAA peaks	Raman shift (cm^−1^)			
	Powder sample	Saline	Serum	Urine
Benzene ring stretch	1620	—	—	—
*ν*C=C	1570	1574	1603	1575
C–C Pyrrole stretch	1553	1554	1594	800, 1554
*γ*C=C in plane	1455	1454	1458	1454
NCC stretch NH bend	1430	1431	1434	1431
Fermi doublet	1360–1340	1360	1363–1342	1360
C–C stretch NH bend	1307	—	—	—
CH bend NH bend	1220–1240	1220–1240	1225	—
Benzene ring breathing	1010	1010	1026	1010
*γ*OH	909	921	996	995
NH bend	881	889	846	—

**Fig. 4:**
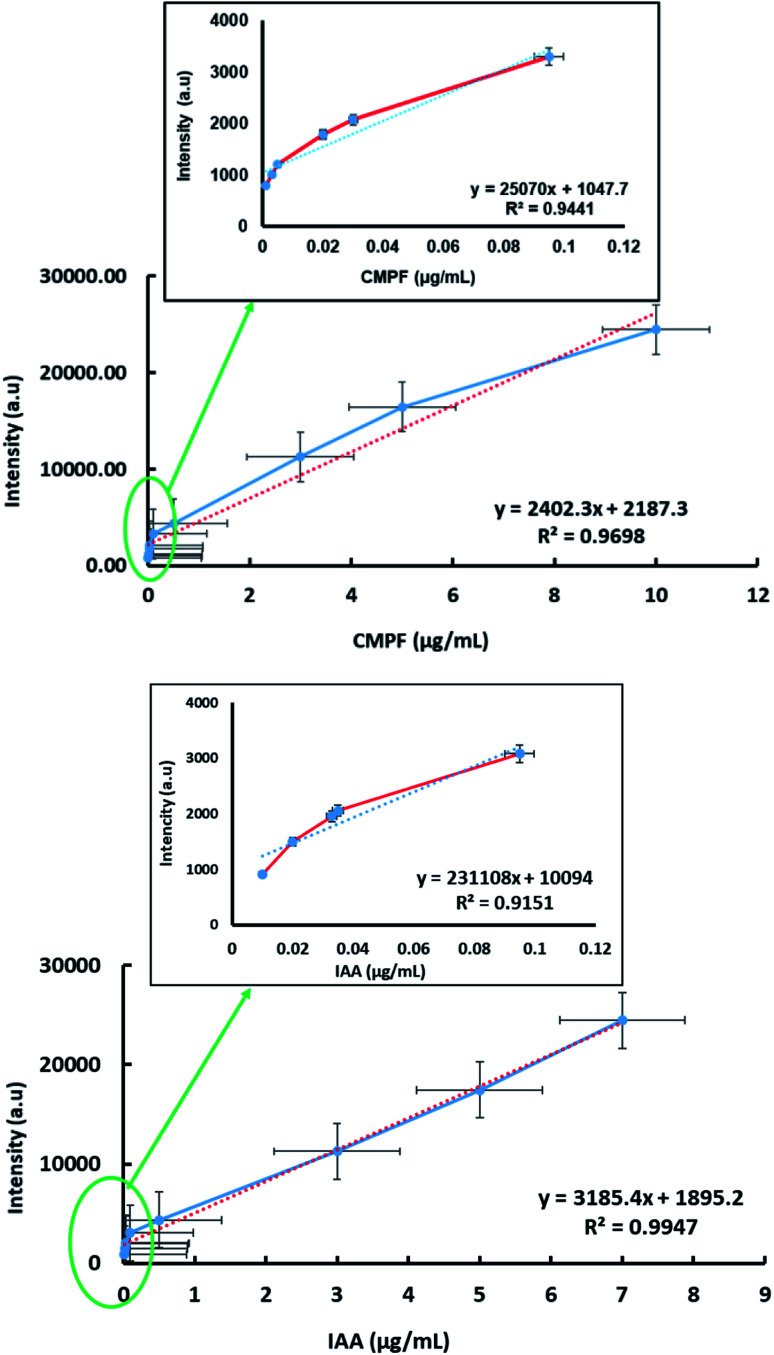
The plot of the linear regions of the concentration-SERS intensity curve at 1260 cm^−1^ as a function of the CMPF concentration (top) and at 1554 cm^−1^ as a function of the IAA concentration (bottom).

The detection of sensitivity and linear dynamic range of the SERS approach were quantified for determining CMPF and IAA at trace levels. Fig. 4 (top) shows a linear relationship in the range from 0.001 to 10 μg mL^−1^ for CMPF with the SERS spectral intensity of the peak at 1260 cm^−1^. Then, we plotted the absolute Raman intensity of the peak at 1260 cm^−1^ with different concentrations of CMPF. The *R*^2^ of the linear regression is 0.969, indicating a high degree of accuracy throughout the assay range. The limit of detection was calculated based on the standard deviation of response and the slope of the calibration curve, and it was 0.04 nM (S/N = 3). The peak intensity at 1554 cm^−1^ was plotted as a function of the IAA concentration from the range of 0.01 to 7 μg mL^−1^ (Fig. 4, bottom). The calibration plots showed linear relationships between the peak intensity and the concentrations of IAA with a correlation coefficient of 0.994. The detection limit of IAA was estimated to be 0.05 μM (S/N = 3).

**Fig. 5:**
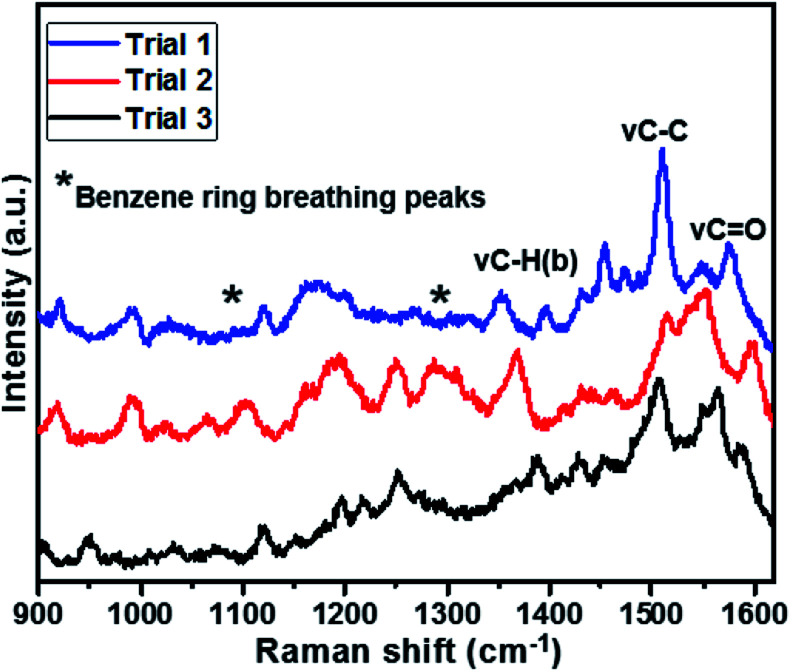
Reproducibility of SERS spectra at 0.02 μg mL^−1^ of IAA.

The reproducibility of the sensor was also studied with the intra-assay. The intra-assay precision of the sensor was evaluated by analysing IAA with a concentration of 0.020 μg mL^−1^ for three times, which exhibited a similar response and the coefficient of variation was 1.7%, as shown in Fig. 5.

### 2.3. Simultaneous detection of CMPF and IAA in serum samples of healthy subjects

**Fig. 6:**
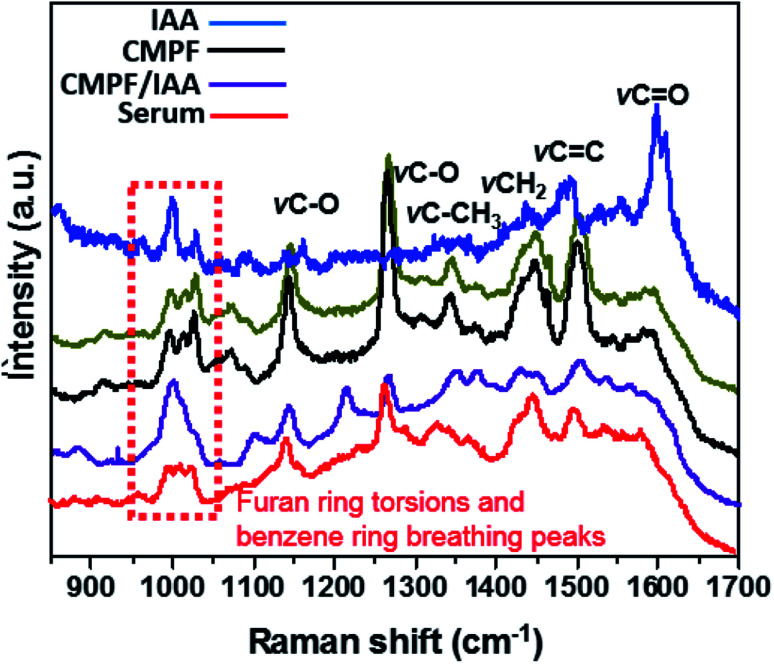
SERS spectra of (i) a mixture of 0.02 μg mL^−1^ CMPF and IAA, (ii) 0.02 μg mL^−1^ CMPF and (iii) 0.02 μg mL^−1^ IAA.

The results of the individual detection of CMPF and IAA molecules from saline and real serum samples have shown non-overlapping Raman bands with limited interference from the biological sample matrix. Keeping this in mind, the specificity of the SERS procedure was utilized for simultaneously detecting CMPF and IAA spiked serum samples collected from healthy subjects. Although serum is a complex matrix, distinct SESR peaks corresponding to CMPF and IAA were obtained (Fig. 6). It should be noted that prominent SERS peaks near 1380 cm^−1^, 1340 cm^−1^ and 1260 cm^−1^ are unique to CMPF and prominent SERS peaks near 1026 cm^−1^, 1594 cm^−1^, 1434 cm^−1^ and 1220–1240 cm^−1^ are unique to IAA.

We compared the sensitivity and analytical time for the modified SERS method of detection with previously reported analytical methods. As shown in Table 3, the detection time of our method was dramatically decreased to be less than 5 min. The optimal time after the addition of the analytes is around 4 min. In addition, our method has more advantages in comparison to the other reported methods such as selectivity and no need for any special scientific instruments. Therefore, the present method is superior in obviating time-consuming sample treatment steps and decreased sample limit. Furthermore, the present method has high sensitivity gained by signal enhancers and it does not require any special scientific instruments.

**Table 3:** Comparison of the performance of the modified SERS *vs.* different methods of detection of IAA

Entry	Method	Matrix	Detection limit/μM	Analytical time^b^	Ref.
1	HPLC (CMMIPs)	Plant tissues	0.025	>12 h	26
2	Differential pulse voltammetry (MWCNT-CS/GCE)	Pea root extracts	0.1	>12 h	27
3	Square wave voltammetry (BDD)	Vegetable samples	1.22	>12 h	28
4	Ampromerty (hemin/rGO/GCE)	Tomato samples	0.074	—a	29
5	Ampromerty (AuNP-GH/GCE)	Phosphate buffer solution	0.21	—a	30
6	Ampromerty (MWCNTs-DHP-GCE)	Plant leaves	0.02	>12 h	31
7	Electrochemical method (PST–rGO)	Plant leaves	0.05	>12 h	32
8	Electrochemical method (carbon tape-modified electrode)	Whole pea seedlings	0.1	<5 min	33
9	Chemiluminescence (trivalent silver)	Human urine, mung bean sprouts and soil samples	4 × 10^−7^	—a	34
10	Photoelectrochemical immunoassay	Plant seeds	2.59 × 10^−7^	—a	35
11	Capillary electrophoresis	Banana, cabbage and cucumber extracts	3.48 × 10^−6^	—a	36
12	Colorimetry	No real sample	7.8 × 10^−2^	—a	37
13	Fluorometry	Fruit juice	6.23 × 10^−5^	—a	38
10	SERS coupled with Au nanoparticles substrate	Human serum and urine	0.05	<5 min	Our study

a The analytical time was not reported.

b Total analytical time included both sample preparation and detection.

### 2.4. Detection of CMPF and IAA in serum samples of CKD patients

**Fig. 7:**
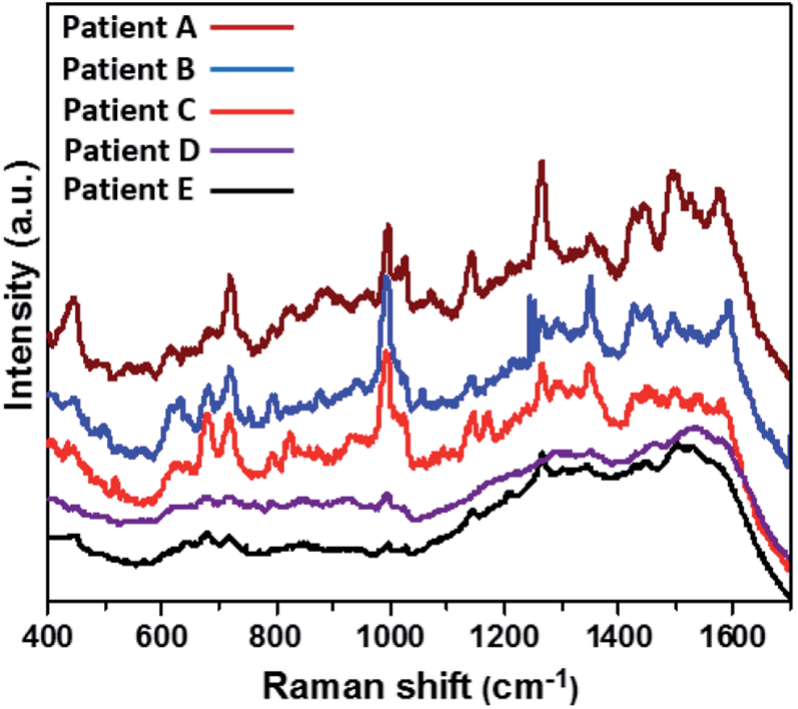
SERS spectra of serum samples collected from CKD patients.

The detection of the individual as well as simultaneous CMPF and IAA molecules from spiked serum samples collected from healthy subjects have been demonstrated. Therefore, this SERS approach was taken a step further for qualitatively and quantitatively checking the presence of both analytes in a more complex serum matrix carrying several CKD patients. Fig. 7 depicts the SERS spectra of unspiked serum samples collected from six uremia patients (A–E). These spectra were recorded from Au-modified SERS substrates to probe the presence of CMPF and IAA clinical uremic toxins *via* the SERS nanotechnology. The presence of SERS peaks are consistent with CMPF, and IAA may be found at 1325–1375 cm^−1^ (*ν*C–CH_3_ vibrations), 1150 and 1275 cm^−1^ (C–O bonds) and 1510 cm^−1^ (C=C bond). These peaks are conspicuous with the spectra of serum samples between patients A and C. However, since this is a more complex matrix, there are also absorption peaks linked with other CKD patients. Some identical characteristic SERS peaks may be due to the presence of other non-PBUT uremic toxins: creatinine (680 cm^−1^) in patient C and uric acid (637 and 1138 cm^−1^) in patients A–C, and urea (1001 and 1045 cm^−1^) in patients A–E. For the results presented within this study, it could be concluded that this SERS approach was capable of the real-time detection of CPMF and IAA in the presence of other clinical uremic toxins within serum samples from uremia patients. However, the failure to obtain completely resolved SERS peaks could be linked with the complexities of the serum matrix as well as the interference of other analytes.

### 2.5. The actual contents of CMPF and IAA in uremic serum samples

Table 4 summarizes the spiked concentration and found concentrations of CMPF and IAA in uremic serum samples.

**Table 4:** The actual contents of CMPF and IAA in uremic serum samples

Sample	Spiked (CMPF), μg mL^−1^	Found, μg mL^−1^	Recovery^a^ (%)	Sample	Spiked (IAA), μg mL^−1^	Found, μg mL^−1^	Recovery^a^ (%)
Patient A	0.001	11.91	109.0	Patient A	0.001	1.26	108.1
Patient B	0.001	10.72	100.1	Patient B	0.001	1.01	102.0
Patient C	0.001	1.97	96.0	Patient C	0.001	0.79	70.7
Patient D	0.001	1.87	90.4	Patient D	0.001	0.55	60.7
Patient E	0.001	1.69	86.2	Patient E	0.001	0.50	58.0

a Recovery = (found concentration− basic concentration)/spiked concentration × 100%.^39^

The detected amounts of two analytes were calculated based on the corresponding calibration curves. A basic concentration of unspiked serum samples was collected from six uremia patients (A–E). The spiked recoveries were calculated using eqn (1).^39^

1 Spike recovery (%) = (found concentration − basic concentration)/spiked concentration × 100%

The average recovery of the patient samples set based on CMPF and IAA was 96.4% and 79.9%, respectively, which suggested that the results are reliable.

## 3. Experimental

### 3.1. Material

**Fig. 8:**
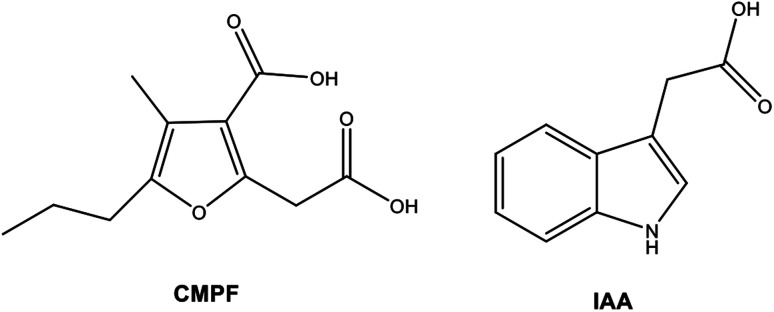
Chemical structure of 3-carboxy-4-methyl-5-propyl-2-furanpropanoic acid (CMPF) and indole-3 acetic acid (IAA).

AuNP-modified SERS substrates in a microscope slide format (RAM-SERS-Au-5) were purchased from Ocean Optics.^40^ 3-Carboxy-4-methyl-5-propyl-2-furanpropanoic acid (CMPF, molecular weight: 240.25 g mol^−1^), and indole-3 acetic acid (IAA, molecular weight: 175.18 g mol^−1^) was provided by Sigma-Aldrich. These chemicals were analytical-grade reagents with more than 98.0% purity and were used as purchased without further purification. The water sample used in the study was Milli-Q® ultrapure water with a resistivity of 18.2 MΩ.cm (at 25 °C). The molecular structures of both biomarkers, CMPF, and IAA, investigated within this study are presented in Fig. 8. Actual serum samples were collected from healthy adult human volunteers.

### 3.2. Measurement by the SERS technique

The Raman spectra of each treated sample were collected at room temperature using a Renishaw Invia Reflex Raman microscope located at the Saskatchewan structural science center (SSSC), University of Saskatchewan. All test samples were prepared by dissolving (1 g) in Milli-Q water within a glass vial. These mixtures were then further diluted to make-up for the required concentrations needed. In order to improve the enhancement factor and the signals, the excitation wavelength and laser power were optimized. An excitation wavelength of 514.5 nm radiation from an argon-ion laser (Modulaser Stellar Pro, UT) and an 1800 l mm^−1^ grating, providing a spectral resolution of approximately 1 cm^−1^, were used. The AuNPs on the glass substrate were premodified with toluene. The surface plasmon resonance of the Au NPs in this medium is between 500 and 600 nm; hence, our choice of 514.5 nm excitation wavelength was optimum. The peaks of surface plasmon resonance are shifted according to the reflective index of the substrate as well as the presence of the modification medium. The Renishaw Invia Reflex Microscope is equipped with several laser excitations along with a software that allows automatic switching of lasers. This instrument is also capable of recording the spectra of samples at varying laser powers. 3.0 mW was the optimum power to obtain the spectra reported in this study. Therefore, the excitation wavelength of 514.5 nm and laser power of 3.0 mW were used. The laser was focused onto the samples using a 100× N PLAN objective (NA = 0.90, Leica Microsystems Inc., Mannheim, Germany), and the backscattered Raman signals were separated by an edge filter and collected with a Peltier cooled CCD detector. Raman spectra were recorded in Streamline™ mode, using a detector time of 60 s, and a pixel resolution of 0.6 μm × 0.6 μm. The spectral position was verified by internal Si (110), which is 520 cm^−1^. A Renishaw Wire (V3.4) was used for data processing and image analysis. We have used the Au-modified SERS for enhancing the spectral acquisition with good flexibility and propensity for minimal SERS signal interference. It satisfies the needed requirements for this analyte detection due to their unique surface roughness, the degree of dispersion and lack of a tendency to aggregate. In addition, AuNP-modified SERS substrates have demonstrated that their testing has shown reliable peak ratio reproducibility.

### 3.3. Preparation of standard solutions of CMPF and IAA in saline and serum samples

Saline samples containing different concentrations of CMPF (0.001, 0.003 and 0.005 μg mL^−1^) and IAA (0.01, 0.02 and 0.035 μg mL^−1^) were first prepared. A stock solution of CMPF and IAA with 2 μg mL^−1^ (w/v) concentration was prepared by dissolving CMPF and IAA powders in 1 mL serum samples. Different concentrations (0.020, 0.030 and 0.095 μg mL^−1^) of CMPF, and (0.010 and 0.050 μg mL^−1^) of IAA solutions were then prepared by serial dilution. In all cases, 10 μL of the sample solutions were dropped on the AuNP-modified SERS substrate for Raman measurement using a Raman microscope. It was allowed to dry up on the surfaces of the SERS substrates before analyzing those dried sample spots (at approximately 0.5 mm diameter). Raman measurements were carried out directly from the coverslip SERS sides, while highly reproducible SERS spectra (of specific sample spots within these dried areas) were recorded as presented within this study.

## 4. Conclusions

In summary, we have used SERS coupled with the Au nanoparticles substrate as an effective, simple and rapid approach for the determination of CMPF and IAA in saline and human serums. The two major novelties and advantages of this method are: (1) to the best of our knowledge, this is the first time that the SERS technique is used for the analysis of PBUTs in biological samples and (2) highly-sensitive quantitative analysis in a very short time (less than 5 min). Furthermore, this method has more advantages in comparison to the other reported methods, such as no need for any complicated sample preparation, the cumbersome analysis process and sophisticated analytical capabilities. This new SERS approach is ultrasensitive, providing a limit of detection for CMPF = 0.04 nM and IAA = 0.05 μM. Due to the non-overlapping nature of narrow Raman bands, SERS was applied for the simultaneous determination of CMPF and IAA in human serum samples. The simultaneous quantitative detection of other protein-bound uremic toxins in the serum and urine of healthy volunteers and patients with CKD is currently underway in our laboratories.

## Ethical statement

Dr Amira Abdelrasoul, the principal investigator of the project, obtained University of Saskatchewan Research Ethics Approval as well as Saskatchewan Health Authority Operational Approval to conduct the research in Saskatchewan Health Authority, in Canada. All experiments were performed in accordance with the governing law. Experiments were approved by the Biomedical Research Ethics Board at University of Saskatchewan. Informed consent were obtained from human participants in this study from the St. Paul Hospital.

## Conflicts of interest

There are no conflicts to declare.

## Supplementary Material
